# Surgeries Performed in Professional Australian Cricketers 2015–2024

**DOI:** 10.1007/s43465-026-01762-w

**Published:** 2026-04-10

**Authors:** John W. Orchard, Anna Saw, Philippa Inge, Kate Beerworth, Leigh Golding, Nick Jones

**Affiliations:** 1 Sydney Medical School, University of Sydney, Sydney, Australia; 2 Cricket Australia, Melbourne, Australia; 3 School of Allied Health, Human Services and Sport, LaTrobe University, Melbourne, Australia

**Keywords:** Arthroscopy, Fracture fixation, Reconstructive surgery, Cricket

## Abstract

**Aim:**

A study describing the distribution and rate of surgical operations performed in professional Australian cricketers, both male and female, over a decade.

**Methods:**

Cricket Australia Athlete Management System was searched for consultations including terms that might reference whether surgery had been performed. Surgeries were any procedure performed in a hospital or day care facility, including non-orthopaedic surgeries.

**Results:**

There were 3419 player years studied between 2015 and 2024, yielding 447 surgeries, a rate of 13.1 surgeries per 100 player years, the majority of which were musculoskeletal (orthopaedic) procedures. The most common procedures were: knee arthroscopy (1.61/100 player years), wrist hand/finger fracture fixations (1.52), shoulder reconstruction (0.91), ankle posterior impingement procedures (0.44) and knee ACL reconstructions (0.41). The rates of surgery in male (12.7) and female (13.5) players were similar. The rate of surgery in male players was lower than a previous audit a decade ago (19.6 which excluded non-musculoskeletal procedures, *P* < 0.001) with the surgeries being performed less often including knee arthroscopy, ankle posterior impingement procedures and shoulder arthroscopy (all *P* < 0.001).

**Discussion/conclusions:**

There is a general consistency in the rate of surgical procedures in professional Australian cricketers, although rates for the recent decade have dropped compared to the one prior in male players. Rates of surgery between male and female cricketers are very similar, and rates are lower than in the football codes in Australia but higher than the general population.

## Introduction

Surgery is known to be commonly undertaken in elite and professional athletes, although there are only a few published audits that take a body-wide overview [1–3]. In the published literature it is far more common to find series of surgeries performed for a particular region, such as knee ACL injuries [4]. In cricket there have been specific audits of hand injuries [5–7], ankle posterior impingements [8–10], facial fractures [11] and lumbar spine surgeries [12–15]. A recent textbook summary of sports medicine specific to cricket contained multiple chapters which referenced surgical procedures in cricket [16].

We aimed to expand on a previous study auditing surgical procedures in elite Australian male cricketers from 2005–2014 inclusive [1]. In particular, we wished to audit the subsequent decade to this previous study to expand to all elite teams in Australia, considering surgical procedure rates for both males and females, and to compare rates from the decade 2015–2024 to the previous decade. Women’s cricket has become fully professional only over the last decade [17] and we feel it is important to audit surgeries in elite female cricketers for the first time.

## Methods

Ethics approval was gained from an Institutional Ethics Committee (La Trobe University HEC20058) which waived the need for individual informed consent. Data was retrieved from Cricket Australia’s online database (Fair Play AMS Pty Ltd). Data included medical records recorded at the time of consultation by team doctors and physiotherapists. Free-text notes were searched for terms potentially relating to surgery: surg, operat*, reconstruct, scope, fixation, repair, ORIF, *otomy, *ectomy, removal, excision, stabilisation, post-op.

Elite Australian cricket players were included if they were contracted to a National, State, Territory, or franchise (Big Bash League) team at the time of surgery. Non-contracted players (e.g., junior pathway) and non-Australian players (e.g., visiting international players contracted to a franchise) were excluded from both numerator and denominator.

Surgeries were considered to be any procedure performed in a hospital or day care facility, including non-orthopaedic surgeries. The procedure required admission and therefore excluded procedures performed at a cricket ground or sports medicine clinic, like suturing of wounds or reduction of minor dislocations. The procedure did not have to be related to cricket, meaning that operations like appendicectomy, tonsillectomy and removal of wisdom teeth were included. Radiological procedures, such as guided injections of cortisone, even if performed in a hospital facility, were not included. Bilateral procedures were considered to be two surgeries.

The primary unit of recording surgical rates was incidence of surgeries (procedures) per 100 player years. If it is considered that players have one season per year (i.e. May-April), then surgeries per 100 player years is the same as 100 player seasons. A rate of 10 surgeries per 100 player years means that in a given year, the approximately 10% of players would have surgery. The rates of surgery are presented as raw rates without including confidence intervals, as they are actual rates of documented occurrence. If we were to predict rates of surgery over the following decade, then we would present these rates followed by confidence intervals.

A comparison was made between the (male) incidence rates in this study and the previous study of surgery in (male only) Australian cricketers a decade earlier. Comparisons were also made between male and female players. Chi-squared tests (in Microsoft Excel) were used to determine significant differences between rates of surgery.

The main ethical restriction was that any surgery with > 0 and < 5 cases could not have the exact number of surgeries stated to preserve confidentiality of players with uncommon conditions.

## Results

There were 3419 player years studied between 2015 and 2024, (Table 1) which followed 406 individual male players and 306 individual female players.

**Table 1:** Calculation exposure (player years of observation)

Season	2015–16	2016–17	2017–18	2018–19	2019–20	2020–21	2021–22	2022–23	2023–24	2024–25	Total
Females	137	158	141	143	149	146	143	133	137	138	1425
Males	193	206	206	208	214	205	208	184	177	193	1994

An initial filter of 79,649 AMS consultations revealed 542 surgeries. There were 108 surgeries excluded, mainly due to being conducted in non-contracted players (there were 4 duplicates and 4 surgeries in international players). There were 13 further surgeries added found through alternate searching, such as other audits.

This exposure finally yielded 447 surgeries, a rate of 13.1 surgeries per 100 player years. (Table 2). If non-musculoskeletal procedures were excluded, the rate of surgeries was 10.3 surgeries per 100 player years. The wrist/hand region had more surgeries than the knee, which was the second most common body area to be operated on.

**Table 2:** Surgeries and rates by body areas

Body area	Procedures	Per 100 player seasons
Wrist/hand	87	2.54
Knee	79	2.31
Shoulder	47	1.37
Ankle	38	1.11
Hip and groin	22	0.64
Foot	21	0.61
Head and neck	11	0.32
Elbow	10	0.29
Lumbar spine	10	0.29
Forearm	8	0.23
Lower leg	7	0.2
Thigh	6	0.18
Trunk and abdominal	< 5	
All surgeries	447	13.07
Musculoskeletal	350	10.24
Non-musculoskeletal	97	2.84
Female	193	13.54
Male	254	12.74

Table 3 reveals that the most common procedures were: knee arthroscopy (1.6 per 100 player years), wrist hand/finger fracture fixations (1.5), shoulder reconstruction (0.9) and hand instability procedures (0.5).

**Table 3:** Rates of specific procedures (where number of procedures >  = 5)

	Indication(s)	Procedure numbers	Per 100 PY
Musculoskeletal procedures			
Knee arthroscopy	Meniscal tears, chondral lesions	55	1.61
Wrist/hand fracture fixation (finger)	Fracture	31	0.91
Wrist/hand fracture fixation (thumb)	Fracture	12	0.35
Wrist/hand fracture fixation (carpal/metacarpal)	Fracture	9	0.26
Shoulder reconstruction	Shoulder instability	31	0.91
Reduction/repair of wrist/hand joint instability	Dislocations, chronic instability	18	0.53
Ankle posterior impingement procedure	Posterior impingement, os trigonum	15	0.44
Knee (ACL) reconstruction	ACL (anterior cruciate ligament) tear	14	0.41
Hip arthroscopy	Labral tear, femoro-acetabular impingement (FAI)	14	0.41
Elbow arthroscopy	Loose bodies, posterior impingement	8	0.23
Shoulder arthroscopy	SLAP (superior labrum anterior posterior) lesion, rotator cuff tear	8	0.23
Hamstring surgery	Repair of grade 3 or 4 hamstring strain/tendon avulsion	6	0.18
Microdiscectomy	Lumbar disc prolapse with nerve impingement	5	0.15
Non-musculoskeletal procedures			
Skin excision (benign lesion)	Diagnostic removal of naevus, solar keratosis	19	0.56
Skin cancer excision	Removal of BCC (basal cell carcinoma) or melanoma	16	0.47
Wisdom teeth removal	Infected or impacted wisdom teeth	12	0.35
ENT (tonsils)	Chronic recurrent tonsilitis	12	0.35
Appendicectomy	Acute or chronic appendicitis	9	0.26
Pterygium surgery (removal)	Pterygium	6	0.18

### Comparison Between Male and Female Players

The rate of surgery in male (12.7) and female (13.5) players was remarkably similar (Table 4). Males had a significantly higher rate of fixation of hand/finger/thumb fractures than females. Females exhibited a trend towards more knee ACL reconstructions being required, although low overall numbers meant that statistical significance was not reached.

**Table 4:** Comparison between male and female player rates of surgery

	Male rate	Female rate	*P* value
Knee arthroscopy	1.5	1.8	0.38
Wrist/hand/finger fracture fixation	1.9	1.0	0.03
Knee reconstruction	0.3	0.6	0.08
Shoulder reconstruction	0.9	0.9	0.98
Ankle posterior impingement procedure	0.5	0.4	0.50
All surgeries	12.7	13.5	0.49

### Comparison Between the Current and Previous Decade in Male Players

The rate of surgery was lower than a previous audit a decade ago (19.6, which only included musculoskeletal procedures) with the surgeries being performed less often including knee arthroscopy, ankle posterior impingement and shoulder arthroscopy (Table 5). There was a trend towards slightly more hand fracture surgery in the recent decade.

**Table 5:** Comparison between male rates of surgery (2015–2024) and the previous decade

	Male rate 2015–2024	Male rate 2005–2014	*P* value
Knee arthroscopy	1.5	3.6	< 0.01
Wrist/hand/finger fracture fixation	1.9	1.2	0.09
Knee reconstruction	0.3	0.5	0.29
Shoulder reconstruction	0.9	0.8	0.87
Ankle posterior impingement procedure	0.5	1.7	< 0.01
Shoulder arthroscopy ± SLAP repair	0.2	1.9	< 0.01
All (non-medical) surgeries	10.2	19.6	< 0.01

### Knee Surgery

The most commonly required procedure was the knee arthroscopy although rates of knee arthroscopy were less than half the rate of a decade ago. Rates of knee ACL reconstruction were similar. Females had slightly higher rate of knee ACL reconstruction than males.

### Shoulder Surgery

Rates of shoulder reconstructions were similar to a decade ago, but rates of shoulder arthroscopy (which included SLAP procedures) dropped substantially. There was a mix of soft tissue (arthroscopic) reconstructions, the most common technique [18], and open (most commonly Latarjet) procedures. There were no open shoulder reconstructions attempted on the bowling arm of any fast bowlers.

### Ankle Posterior Impingement Surgery

Ankle posterior impingement surgeries were the most common procedure of this region but were reduced in rate compared to the previous decade. These were equally common in male players and female players.

### Wrist and Hand Surgery

The most common region for surgery was the wrist and hand [19], and these surgeries were generally unchanged in rate from the previous decade. The majority of procedures were fracture fixations (finger > thumb > hand) followed by procedures to correct instability (either reductions or reconstructions).

### Medical Procedures

Rates for non-injury surgery are presented with common procedures included appendicectomy, tonsillectomy, and removal of wisdom teeth. These procedures were expected as quite common procedures in young adults in the general population. We also noted a consistent rate of procedures to remove skin cancers and eye pterygiums, with perhaps these procedures having some relation to the propensity for cricketers to spend long period of time outdoors.

## Discussion

This study shows that the rate of surgery in elite cricketers in Australia is decreasing slightly. The rate of surgery in elite male and female cricketers is very similar. Males miss slightly more playing time through injury than females, which is presumed to be due to the fixturing of more red ball cricket, but overall injury incidence is similar [20]. It appears that access to surgery is similar between male and female players in Australia.

Our study does have some weaknesses and limitations. The methods of auditing are subject to error. That is, a case or two in each category could possibly have been missed (or an ineligible case included) but the overall figures are likely to be quite accurate.

The most common procedure from this series, knee arthroscopy [21], has been performed less in male cricketers over the past decade compared to the one prior. In recent years the success of knee arthroscopy in middle aged patients been questioned due to the results of randomised control trials (RCTs) [22, 23]. There have not been RCTs for knee arthroscopy in the elite athletic population, so it can neither be presumed that the results of such trials would be poor nor successful. However the previous presumption that knee arthroscopy is best practice management for most symptomatic cartilage lesions in the elite athlete is now more commonly questioned, and perhaps explains declining rates. There are small improvements associated with knee arthroscopy compared to sham surgery which might be enough to justify the surgery in elite athletes but not the general population, as marginal gains are presumably more valuable for elite athlete [23].

Shoulder arthroscopy (which included SLAP procedures) is another procedure that has recent reviews questioning the evidence base [24] where we also saw reducing rates over the past decade in elite cricketers. However we also found declining rates of ankle posterior impingement surgery. Posterior impingement surgery has been subjected to RCTs to compare open and arthroscopic procedures [25] but there has not ever been an RCT between posterior impingement surgery and conservative treatment. Therefore, it probably represents more of a judgement call in recent years that not all cases of posterior impingement surgery with a prominent process or os trigonum require surgery, even though surgery is still regularly performed after failure of conservative management [26].

There have been few other audits in elite sport of other varieties to compare to. Rates of surgery in rugby league players in Australia (35.6 procedures per 100 players years in the 2000s) [3] and professional Australian footballers in 2004 (30 procedures per 100 player years) [27] appear to have been previously higher than cricketers.

A publication of the rates of surgery in College (elite amateurs aged 18–22) USA athletes published rates expressed in terms of surgeries per 10,000 athletic exposures (AEs, matches and training sessions are an exposure each) [2]. If it was assumed that athletes average 100 exposures per year, then the rates could be comparable to our incidence rate of surgeries per 100 player years. The highest rates reported were women’s gymnastics (8.86 per 10,000 AEs), men’s football (6.07), men’s wrestling (5.32) and women’s basketball (4.59). They also found similar rates of surgery between male and female athletes [2]. Across US college sport, knee ACL reconstruction, knee arthroscopy and shoulder reconstruction were the most common surgeries performed. Rates of hand fracture fixation were relatively lower than in cricket (in terms of relative to other procedures).

There are also few comparative publications in which to compare age-matched rates of surgery in the general population. In 2001, the average Australian from age 20–35 years only underwent 2.2–2.3 musculoskeletal procedures per 100 patient years [28], a lower rate than the elite cricketers in this study. The overall surgical procedure rate for young Australians was 8 procedures per 100 patient years in 2001, which is closer but still lower than the rate we have reported [28]. Musculoskeletal procedures were only the second most common category of surgeries for the general population of this age range, with the most common (by far) being obstetric and gynaecological procedures (in females only) [28]. The preponderance of obstetric and gynaecological procedures in this age group in the general population means that females between 20 and 35 have higher rates than males of this age in the general population. It can be stated that elite cricketers have higher rates of musculoskeletal procedures than the general Australian population of the same age, but that they also have lower rates of musculoskeletal procedures than professional football players.

In conclusion, this study has shown a rate of musculoskeletal surgeries in cricketers that is higher than the general population but lower than football players, with rates similar for females as per males. This is the first study documenting rates of surgery in elite female cricketers. Knee arthroscopy is still the most common procedure but with a declining rate. Finger fracture fixation procedures are also particularly common.

## Data Availability

De-identified data for this project would be available on request from the authors for reasonable use, such as potential pooling for a meta-analysis. Ethical approval did not include storage in a data repository.
